# The relationship between body mass index and perceived control over labor

**DOI:** 10.1186/s12884-023-06063-w

**Published:** 2023-10-25

**Authors:** Anna R. Whelan, Brock E. Polnaszek, Olivia Recabo, Melissa A. Clark, Adam K. Lewkowitz, Nina K. Ayala

**Affiliations:** 1 Department of Obstetrics and Gynecology, Division of Maternal-Fetal Medicine, Women &, Infants Hospital of Rhode Island, Alpert Medical School of Brown University, 101 Dudley St, Providence, RI 02905 USA; 2 Department of Obstetrics and Gynecology, Division of Maternal-Fetal Medicine, University of Massachusetts Chan Medical School, Worcester, MA USA; 3 Department of Obstetrics and Gynecology, New York Medical College, Valhalla, NY USA

**Keywords:** Control, Labor agentry, Patient experience, Weight bias, Weight stigma

## Abstract

**Background:**

*Individuals with an increased* body mass index (BMI) (≥ 30 kg/m2) experience higher rates of perinatal mental health disorders than individuals with BMI < 30. Personal experience of decreased control over labor has been associated with the development postpartum mood and anxiety disorders. However, no studies have investigated the association between BMI and experience of control over labor. This study aimed to assess perceived control over labor and compare patients with BMI ≥ 30 to those with BMI < 30.

**Methods:**

We performed a secondary analysis of a cross-sectional study of postpartum patients who delivered at term (37–41 weeks gestation). Postpartum, participants completed the Labour Agentry Scale (LAS), a validated tool to assess perceived control over labor/birth. Demographic, maternal health history and obstetric/neonatal outcomes were abstracted from the patient chart. Bivariate analyses were performed between those with BMI < 30 and those with BMI ≥ 30 using Fisher’s exact test. Continuous LAS scores were compared between patients with BMI < 30 and BMI ≥ 30 using Wilcoxon rank-sum tests. Higher LAS scores indicate higher perceived control over labor. Multivariable linear regression was then performed to account for confounding factors identified a priori.

**Results:**

There was no difference in LAS between those with BMI ≥ 30 and BMI < 30. When stratified by World Health Organization (WHO) class of BMI, those with BMI ≥ 40 had a significantly lower LAS scores than those with BMI < 30 (147 vs. 163, *p* = 0.02), however, this finding was no longer significant after controlling for length of labor and cesarean birth.

**Conclusion:**

Only participants with the highest BMI experienced decreased control over labor, and this finding was no longer significant after controlling for mode of delivery and length of labor. Further research into the experience of birthing people with BMI ≥ 30 is critical to understand the increased risk of perinatal mood disorders among this population.

## Background

Perinatal mental health disorders affect 1 in 5 birthing persons and are the most common complication of childbirth [1, 2]. Individuals with body mass index ≥ 30 k/m^2^ are at an increased risk for perinatal mental health disorders [3, 4]. People with BMI ≥ 30 are 50% more likely to have antenatal depression, 40% more likely to have postpartum depression, and 25% more likely to develop postpartum anxiety compared to those with BMI < 30 kg/m^2^ [3–8]. The exact etiology of these increased risks has not yet been elucidated.

Patient experience of labor and birth has been associated with the development of postpartum mental health disorders. For example, individuals who undergo unplanned cesarean births experience increased rates of postpartum depression and anxiety [9–11]. The Labour Agentry Scale (LAS) is a validated questionnaire which assesses an individual’s feelings of control over their birth experience [12]. Lower scores on the LAS are associated with an increase in the risk of postpartum mental health disorders [13].

The time immediately surrounding parturition is an incredibly vulnerable time for many patients [14], but particularly for those with a high BMI. People with a high BMI experience increased rates of medical intervention, as well as problems with fetal monitoring, higher risk surgical procedures and interaction with a larger volume of healthcare workers [15–17]. This may lead to individuals feeling a decrease in their sense of control over labor and their bodies.

To our knowledge, there have been no studies to date which specifically evaluate patient experience of control over labor based on their body mass index as a potential etiology of increased risk for perinatal mental health disorders. This study aimed to assess perceived control over the birth process and compare patients with BMI ≥ 30 to those with BMI < 30.

## Methods

We performed a secondary analysis of a cross-sectional survey study of patients admitted to the labor and delivery unit at a single academic medical center, during the months of June and July 2021 [18]. This study was approved by the institutional review board (#1691795). English-speaking, nulliparous patients with singleton, non-anomalous pregnancies at gestational age ≥ 37 weeks gestation were eligible for inclusion in our study. Participants were ineligible if they were scheduled for cesarean delivery (CD) or had a contraindication to labor.

After obtaining written consent, participants filled out a questionnaire which included the Labour Agentry Scale (LAS), a validated instrument that assesses childbirth control [12]. The LAS consists of 29 items for which participants answer using a 7-point Likert scale [12]. Higher scores denote experiencing more control over labor. Median scores on the LAS for the control group in the ARRIVE trial (*N* = 3037) birthing people ranged from 164 (interquartile range (IQR) 143–181) [19]. For this secondary analysis, the primary outcome was LAS score, and participants were stratified by body mass index (BMI) at the time of delivery admission – calculated by stated or measured weight in kilograms divided by height in meters-squared). BMI was first dichotomized into two groups—participants with BMI ≥ 30 kg/m^2^ versus those with BMI < 30 kg/m^2^—and then into multiple groups so that BMI < 30 was compared to each WHO class of obesity (class I BMI 30–34.9, class II BMI 35–39.9, class III BMI ≥ 40) [20]. Demographics (including age, race, ethnicity), maternal medical history (gestational and pregestational diabetes, hypertensive disorders of pregnancy), obstetric interventions (induction of labor, mode of delivery, anesthesia, duration of labor) and neonatal outcomes (NICU admission, antibiotic administration, phototherapy) were extracted from the electronic medical records by trained researchers and compared between BMI groups. We hypothesized that individuals with a BMI ≥ 30 kg/m^2^ experience less control over labor than individuals with BMI < 30 kg/m^2^.

Data were analyzed using STATA v.15 (College Station, TX). Fisher’s exact test was performed for categorical variables and Wilcoxon Rank-sum for continuous variables. The primary outcome of score on the LAS was compared between those BMI < 30 kg/m^2^ and those with BMI ≥ 30 kg/m^2^ as well as those without obesity to those with WHO class of obesity as well (class I BMI 30–34.9, class II BMI 35–39.9, class III BMI ≥ 40) compared with BMI < 30 kg/m^2^. Multivariable linear regression was performed controlling for a priori confounders of mode of delivery and labor length [18].

## Results

Of 149 participants in the original study, 148 had data available for BMI and 87 (58.4%) reported a BMI ≥ 30. There were no differences in maternal age at delivery or participant stated race/ethnicity with the majority of participants identifying as white between those with a BMI ≥ 30 and those with BMI < 30 (Table 1). There were also no differences in the proportion of patients with medical or psychiatric comorbidities between those with BMI ≥ 30 and those with BMI < 30.

**Table 1:** Demographic and delivery characteristics among pregnant people with body mass index < 30 compared with ≥ 30 kg/m2

	BMI < 30 (*N* = 61)	BMI ≥ 30 (*N* = 87)	*P*-value
Maternal Age, median (IQR)	30 (26–33)	28 (24–31)	0.09
Patient-reported race/ethnicity			0.62
Black	2 (3.3)	6 (6.9)	
Latina	8 (13.1)	18 (20.7)	
American Indian/Indigenous	2 (3.3)	3 (3.4)	
Asian/Pacific Islander	1 (1.6)	1 (1.2)	
White	48 (78.7)	59 (67.8)	
Indication for admission			0.15
Labor	39 (63.9)	43 (49.4)	
IOL (scheduled)	17 (27.9)	29 (33.3)	
IOL (unplanned)	5 (8.2)	15 (17.3)	
Length of labor (hours), median (IQR)	15 (9–21)	19 (12–31)	0.02
Mode of delivery^a^			0.07
SVD	46 (75.4)	52 (59.8)	
OVD	4 (6.6)	4 (4.6)	
CD	11 (18.0)	31 (35.6)	
NICU admission	6 (9.8)	7 (8.2)	0.77
Neonatal therapy^b^	7 (11.5)	13 (14.9.9)	0.63

Data are N(%) unless otherwise stated. Significance at *p* < 0.05

Fisher’s exact and Wilcoxon Ranksum tests used for analysis

*IQR* interquartile range, *IOL* induction of labor, *SVD* spontaneous vaginal delivery, *OVD* operative vaginal delivery, *CD* cesarean delivery

^a^OVD consists of both forceps-assisted and vacuum-assisted deliveries

^b^Neonatal therapy includes the need for supplemental O2, phototherapy for jaundice, neonatal antibiotics

The majority of participants underwent spontaneous vaginal delivery (75.4% for patients with BMI < 30, 59.8% for patients with BMI ≥ 30) and there were no differences in mode of delivery between groups. However, those with BMI ≥ 30 had significantly longer median labor time from admission to delivery than those with BMI < 30 (15 h vs. 19 h, *p* < 0.02). There was no difference in rate of NICU admission or need for neonatal therapy between groups (Table 1).

**Fig. 1:**
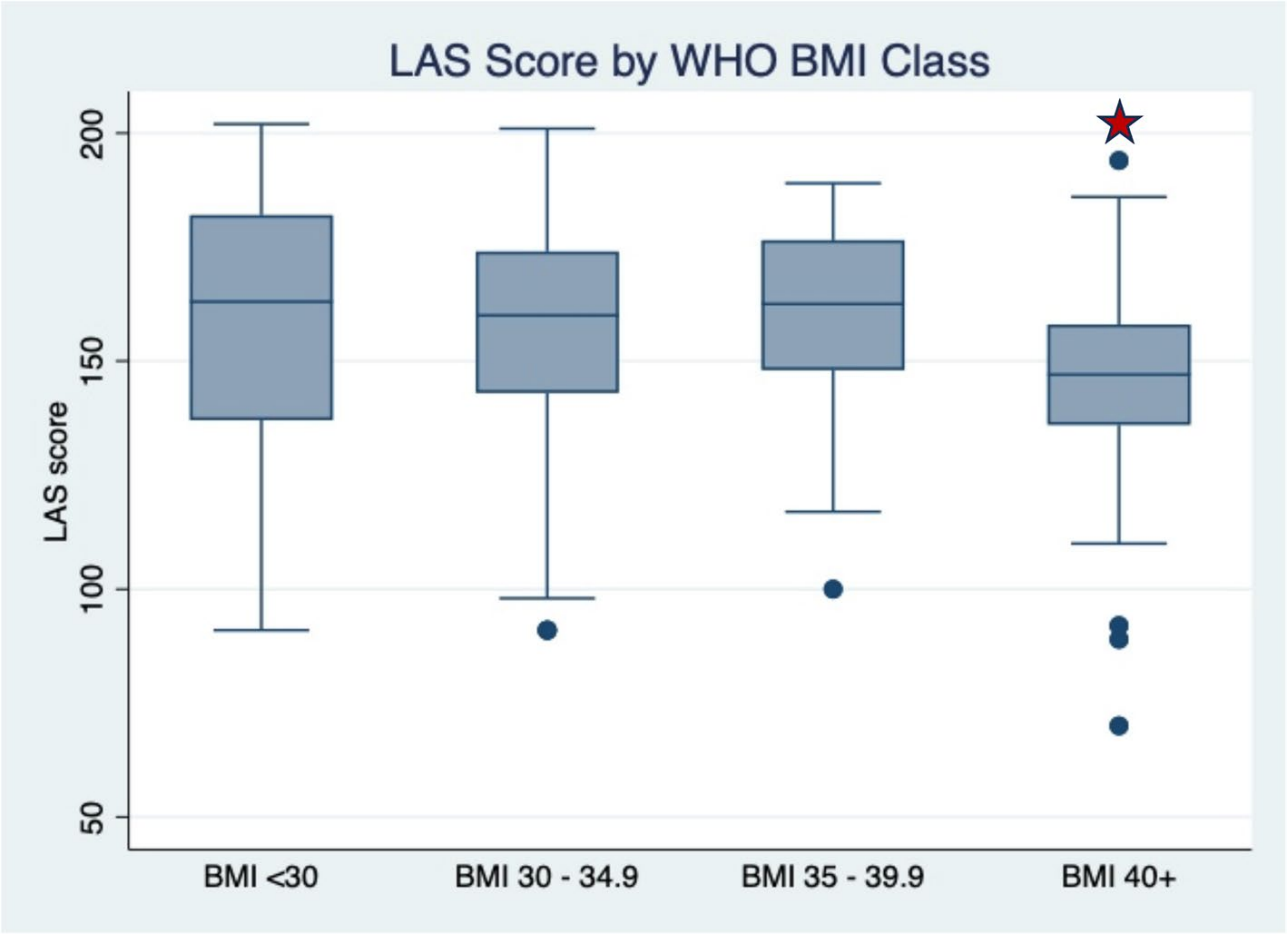
LAS score by WHO BMI class. Star indicates significance at *p* < 0.05 when compared to BMI < 30

There were no significant differences in scores on the LAS for those who had BMI < 30 compared with BMI ≥ 30 (163 vs 154, *p* = 0.11). When analyzed by BMI category with BMI < 30 as the reference, those with BMI ≥ 40 had significantly lower LAS scores (163 vs 147, *p* < 0.02) (Fig. 1). However, after controlling for mode of delivery and labor duration, BMI was no longer significantly associated with differences in LAS score (Table 2).

**Table 2:** Labour agentry scale scores by obesity status, obesity class and multivariable linear regression

	Non-obese BMI < 30 (*N* = 61)			Obese BMI ≥ 30 (*N* = 87)		*P*-value	
Total LAS						0.11	
Median (IQR)	163 (137–182)			154 (141–174)			
By Class of Obesity							
	Non-obese BMI < 30 (reference) *N* = 61)	Class I BMI 30–34.9 (*N* = 37)	*P*-value	Class II BMI 35–39.9 (*N* = 20)	*P*-value	Class III BMI ≥ 40 (*N* = 30)	*P*-value
Total LAS			0.34		0.45		0.02
Median (IQR)	163 (137–182)	163 (143–174)		162.5 (148–176.5)		147 (136–158)	
Multivariable Linear Regression							
	Coeff (SD)			t		95%CI	
Constant	166.43 (3.81)						
BMI > 40	-2.45 (4.39)			-0.56		-11.12,6.23	
Cesarean delivery	-16.13 (4.85)			-3.32		-25.71,-6.45	
Duration of labor (hrs)	-0.22 (0.12)			-1.84		-0.45,0.02	

Significance at *p* < 0.05

Wilcoxon Ranksum test used for analysis of LAS score between non-obese and obesity participants as well as between categories of obesity and non-obese

## Discussion

In this study, no significant differences were identified between experience of control over labor as measured by LAS scores between patients with a BMI < 30 kg/m^2^ vs ≥ 30 kg/m^2^. While patients in the highest category of BMI (≥ 40) had significantly lower LAS scores, this was ultimately not significant in the multivariable analysis.

We hypothesized that patients with a high BMI would experience less control over labor due to the increased need for intervention, operative delivery and prolonged labor [15, 16, 21, 22]. However, despite significantly longer labors, we did not find a difference in experience between the two groups. Potential explanations for this lack of significant difference between groups include the relatively small sample size and the racially homogeneous cohort.

Additionally, we did not detect a difference in mode of delivery among those with BMI ≥ 30 compared to patients with BMI < 30 [21–23]. This may be a type II error due to our small sample size. As cesarean delivery could potentially explain the difference in LAS scores between patients with BMI > 40 compared to BMI < 30, a larger study is needed to confirm these findings.

Understanding the increased rates of perinatal mental health disorders among patients with a high BMI is of the utmost importance, as perinatal mental health disorders are the most common cause of maternal morbidity and mortality in the United States [24]. Larger studies are needed to confirm the lack of significant difference in birth experience between patients with high BMIs and those with BMI < 30. Furthermore, qualitative research is planned to help further elucidate causes and areas for intervention to reduce the risk of perinatal mental health disorders in this population.

Our study was consistent with prior research which utilized the LAS. Median scores for participants with BMI < 30 was 163 (IQR 137–182), which is comparable to the control group from the ARRIVE trial (median 164, IQR 143–181) [19]. Additionally, the difference in length of labor between those with BMI < 30 and those with BMI ≥ 30 demonstrated in the current study is consistent with findings from Hilliard et al., who reported a difference in length of labor of approximately 3 h between groups [15].

Our study was limited by small sample size, which may increase the likelihood of type II error when analyzing differences between study groups. BMI was also calculated from patient stated weight and height on admission to labor and delivery, which may have led to measurement error as patients regularly underestimate/underreport weight [25]. Nevertheless, this study has many strengths. To our knowledge, this if the first study to assess patient experience of control over labor and how that interacts with patient BMI. The detailed extraction of data from the medical records allowed us to assess and control for differences between those with a high BMI and those with BMI < 30 kg/m^2^.

## Conclusion

No significant differences in patient experience of control over labor were found between birthing individuals with a BMI ≥ 30 kg/m^2^ and those with BMI < 30 kg/m^2^. Larger studies are planned to confirm these findings as well as qualitative studies on obstetric patient experience among patients with high BMI is necessary in order to understand the increase in rates of perinatal mental health disorders in this population.

## Data Availability

The datasets used and/or analysed during the current study available from the corresponding author on reasonable request.
